# Development and Validation of a Nomogram for Predicting Survival in Male Patients With Breast Cancer

**DOI:** 10.3389/fonc.2019.00361

**Published:** 2019-05-14

**Authors:** Siying Chen, Yang Liu, Jin Yang, Qingqing Liu, Haisheng You, Yalin Dong, Jun Lyu

**Affiliations:** ^1^Department of Pharmacy, the First Affiliated Hospital of Xi'an Jiaotong University, Xi'an, China; ^2^Clinical Research Center, the First Affiliated Hospital of Xi'an Jiaotong University, Xi'an, China; ^3^School of Public Health, Xi'an Jiaotong University Health Science Center, Xi'an, China

**Keywords:** male breast cancer, nomogram, breast cancer specific survival, C-index, AJCC stage

## Abstract

Male breast cancer (MBC) is rare, and most patients are diagnosed at an advanced stage. We aimed to develop a reliable nomogram to predict breast cancer-specific survival (BCSS) for MBC patients, thus helping clinical diagnosis and treatment. Based on data from the Surveillance, Epidemiology, and End Results (SEER) database, 2,451 patients diagnosed with MBC from 2010 to 2015 were selected for this study. They were randomly assigned to either a training cohort (*n* = 1715) or a validation cohort (*n* = 736). The Multivariate Cox proportional hazards regression analysis was used to determine the independent prognostic factors, which were then utilized to build a nomogram for predicting 3- and 5-year BCSS. The discrimination and calibration of the new model was evaluated using the Concordance index (C-index) and calibration curves, while its accuracy and benefits were assessed by comparing it to the traditional AJCC staging system using the net reclassification improvement (NRI), the integrated discrimination improvement (IDI), and the decision curve analysis (DCA). Multivariate models revealed that age, AJCC stage, ER status, PR status, and surgery all showed a significant association with BCSS. A nomogram based on these variables was constructed to predict survival in MBC patients. Compared to the AJCC stage, the C-index (training group: 0.840 vs. 0.775, validation group: 0.818 vs. 0.768), the areas under the receiver operating characteristic curve of the training set (3-year AUC: 0.852 vs. 0.778, 5-year AUC: 0.841 vs. 0.774) and the validation set (3-year AUC: 0.778 vs. 0.752, 5-year AUC: 0.852 vs. 0.794), and the calibration plots of this model all exhibited better performance. Additionally, the NRI and IDI confirmed that the nomogram was a great prognosis tool. Finally, the 3- and 5-year DCA curves yielded larger net benefits than the traditional AJCC stage. In conclusion, we have successfully established an effective nomogram to predict BCSS in MBC patients, which can assist clinicians in determining the appropriate therapy strategies for individual male patients.

## Introduction

Breast cancer in men is an uncommon disease, accounting for approximately 1% of all breast cancers and <1% of all cancers in men (1, 2). The lifetime risk of male breast cancer (MBC) is nearly 1:1000, compared to 1:8 for a woman (2). Although male patients account for only a small proportion of breast cancer patients, the incidence of MBC continues to increase by 1.1% annually, and the reported mortality rates are comparable to those in women (3, 4). Increasing evidence indicates that MBC may be different from female breast cancer (FBC), with unique molecular subtypes (5, 6). Therefore, MBC should be considered as a separate disease and should not be treated according to the guidelines for FBC.

The risk factors for MBC are different from those for FBC (7). Furthermore, the gene polymorphism seen in basal-like cancer with *BRCA1* mutation in FBC is not frequently presented in MBC (8). However, BRCA2 mutation is more frequent in MBC and is seen in 4–16% of the cases and may be associated with poor survival (4). MBC is often diagnosed at a more advanced stage than FBC, which could be attributed to the lack of symptoms at initial presentation as well as its unknown biology (9, 10). Previous studies have shown that (a) mean age at diagnosis of MBC is 5–10 years more than that for FBC, and (b) the lymph node status is a significant prognostic factor of overall survival in patients with MBC (11, 12). Additionally, studies have identified higher rates of estrogen receptor (ER) positivity in MBC compared to FBC (12).

Clinically, several differences exist between MBC and FBC, in terms of the biological characteristics, hormone levels, and risk factors (7, 13). Therefore, distinct therapeutic approaches and management strategies are required for MBC. Nomograms, which are reliable and convenient prognostic tools, have been widely used to predict specific outcomes in clinical oncology. They can quantitatively predict the prognosis in certain patients using known and vital prognostic factors, and illustrate the numerical probability of clinical outcomes (14, 15). The American Joint Committee for Cancer (AJCC) staging system is a tool commonly used by oncologists to predict disease progression and design therapeutic strategies (16, 17). However, given the various factors that influence the course of cancer, prognosis based on the AJCC staging alone is unreliable. This study aims to establish a comprehensive prognostic evaluation of MBC by building a nomogram to understand the risk factors and prognosis better. We also propose to compare the prognostic value of the nomogram with that of the AJCC staging system based on patient data available in the Surveillance, Epidemiology, and End Results (SEER) database.

## Materials and Methods

### Patient Selection and Data Processing

Patient data were screened from the SEER database (covering 18 registries) using the latest SEER^*^ Stat version 8.3.5 (https://seer.cancer.gov/). We initially excluded other cancer diagnosis and selected 2,983 male patients over 18 years of age who were diagnosed with breast cancer between 2010 and 2015. The following variables were evaluated: age, race, marital status, histology, grade, AJCC stage, metastatic sites, ER status, progesterone receptor (PR) status, human epidermal growth factor 2-neu (HER2) status, surgery, radiation, chemotherapy, follow-up time, cancer-specific death, and all-cause death. We excluded patients who were diagnosed at autopsy or by death certificate (*n* = 15), as well as those who did not have complete information on all the above variables (race unknown: *n* = 21, grade unknown: *n* = 259, stage unknown: *n* = 69, metastatic sites unknown: *n* = 29, ER unknown: *n* = 54, PR unknown: *n* = 15, HER2 unknown: *n* = 46, surgery unknown: *n* = 16, and radiation unknown: *n* = 8). Ultimately, we identified 2,451 eligible patients for our study. All data from the SEER database was free, and this study was approved by the Institutional Research Committee of the First Affiliated Hospital of Xi'an Jiaotong University.

### Nomogram Development and Statistical Analyses

For nomogram construction and validation, we randomly divided all the patients into training (*n* = 1715) and validation (*n* = 736) cohorts in a ratio of 7:3 (18, 19). Multivariate Cox proportional hazards regression analysis was performed to identify variables (*P* < 0.05) that significantly affected breast cancer-specific survival (BCSS) and overall survival (OS) in the training group. Using these identified prognostic factors, we constructed a nomogram for predicting 3- and 5-year survival rates in MBC patients.

The nomogram was validated internally in the training cohort and externally in the validation cohort. To evaluate the discriminative ability of the nomogram, we used the concordance index (C-index) and the receiver operating characteristic curve (ROC) and assessed the area under the curve (AUC) (20, 21). The calibration curves were used to compare the association between the actual outcomes and the predicted probabilities (22). Both discrimination and calibration were evaluated using bootstrapping with 1,000 resamples. To compare the accuracy of the new model with that of the traditional AJCC staging model, the net reclassification improvement (NRI) and the integrated discrimination improvement (IDI) were determined (23). The clinical usefulness and benefits of the predictive model were estimated by decision curve analyses (DCA) (24).

All statistical analyses were performed using the SPSS 24.0 (SPSS Inc., Chicago, IL, USA) and the R software (version 3.4.3; http://www.r-project.org/). A *P* < 0.05 was considered to be statistically significant.

## Results

### Patient Characteristics

We evaluated a total of 2451 MBC patients from 2010 to 2015. The training and validation cohorts consisted of 1,715 and 736 cases, respectively, selected by the random split-sample method (split ratio: 7:3). In the training cohort, the majority of patients were over 65 years old (61.0%), white (80.7%), and married (65.7%). Moreover, ductal carcinoma was the most common histopathologic type of MBC, and grades II and III of tumor differentiation degree accounted for 52.7 and 35%, respectively of all the cases. In patients with metastatic MBC, although the incidence of bone metastases was the highest, they were seen in only 4.3% of the cases. A large proportion of the patients were positive for ER (97.1%), positive for PR (90.7%), and negative for HER2 (84.8%). Besides, most of the MBC patients had undergone surgery, and the median follow-up time for both the sets were both 27 months. Table 1 presents the detailed information for the validation and training cohorts, which were comparable.

**Table 1:** Patients' demographics and clinicopathological characteristics.

**Characteristics**	**Total cohort**	**Training cohort**	**Validation cohort**	***P* value**
	2,451 (100%)	1,715 (70.0%)	736 (30.0%)	
**Age**				0.627
<65	947 (38.6%)	668 (39.0%)	279 (37.9%)	
≥65	1,504 (61.4%)	1,047 (61.0%)	457 (62.1%)	
**Race**				0.605
White	1,984 (80.9%)	1,384 (80.7%)	600 (81.5%)	
Black	351 (14.3%)	245 (14.3%)	106 (14.4%)	
Other	116 (4.7%)	86 (5.0%)	30 (4.1%)	
**Marital status**				0.121
Married	1,619 (66.1%)	1,126 (65.7%)	493 (67.0%)	
Unmarried	700 (28.6%)	505 (29.4%)	195 (26.5%)	
Unknown	132 (5.4%)	84 (4.9%)	48 (6.5%)	
**Histology**				0.518
Ductal	2,264 (92.4%)	1,588 (92.6%)	676 (91.8%)	
Lobular	23 (0.9%)	16 (0.9%)	7 (1.0%)	
Mixed ductal and lobular	45 (1.8%)	34 (2.0%)	11 (1.5%)	
Others	119 (4.9%)	77 (4.5%)	42 (5.7%)	
**Grade**				0.223
I	305 (12.4%)	210 (12.2%)	95 (12.9%)	
II	1,294 (52.8%)	903 (52.7%)	391 (53.1%)	
III	848 (34.6%)	601 (35.0%)	247 (33.6%)	
IV	4 (0.2%)	1 (0.1%)	3 (0.4%)	
**AJCC stage**				0.289
I	861 (35.1%)	597 (34.8%)	264 (35.9%)	
II	1,038 (42.4%)	724 (42.2%)	314 (42.7%)	
III	403 (16.4%)	296 (17.3%)	107 (14.5%)	
IV	149 (6.1%)	98 (5.7%)	51 (6.9%)	
**Bone metastasis**				0.398
Yes	112 (4.6%)	74 (4.3%)	38 (5.2%)	
No	2,339 (95.4%)	1,641 (95.7%)	698 (94.8%)	
**Brain metastasis**				0.070
Yes	6 (0.2%)	2 (0.1%)	4 (0.5%)	
No	2,445 (99.8%)	1,713 (99.9%)	732 (99.5%)	
**Liver metastasis**				0.301
Yes	17 (0.7%)	10 (0.6%)	7 (1.0%)	
No	2,434 (99.3%)	1,705 (99.4%)	729 (99.0%)	
**Lung metastasis**				0.039
Yes	56 (2.3%)	32 (1.9%)	24 (3.3%)	
No	2,395 (97.7%)	1,683 (98.1%)	712 (96.7%)	
**ER status**				0.789
Positive	2,383 (97.2%)	1,666 (97.1%)	717 (97.4%)	
Negative	68 (2.8%)	49 (2.9%)	19 (2.6%)	
**PR status**				0.574
Positive	2,229 (90.9%)	1,556 (90.7%)	673 (91.4%)	
Negative	222 (9.1%)	159 (9.3%)	63 (8.6%)	
**HER2 status**				0.507
Positive	284 (11.6%)	207 (12.1%)	77 (10.5%)	
Negative	2,088 (85.2%)	1,454 (84.8%)	634 (86.1%)	
Borderline	79 (3.2%)	54 (3.1%)	25 (3.4%)	
**Surgery**				0.753
Yes	2,310 (94.2%)	1,618 (94.3%)	692 (94.0%)	
No	141 (5.8%)	97 (5.7%)	44 (6.0%)	
**Radiotherapy**				0.679
Yes	670 (27.3%)	473 (27.6%)	197 (26.8%)	
No	1,781 (72.7%)	1,242 (72.4%)	539 (73.2%)	
**Chemotherapy**				0.686
Yes	911 (37.2%)	633 (36.9%)	278 (37.8%)	
No	1,540 (62.8%)	1,082 (63.1%)	458 (62.2%)	
**Median follow-up time (Months, 25th−75th percentile)**	27 (12–46)	27 (13–45)	27 (12–49)	0.735

*AJCC, The American Joint Committee for Cancer; ER, Estrogen receptor; PR, Progesterone receptor; HER2, Human epidermal growth factor 2-neu*.

### Screening for Prognostic Factors for BCSS

Based on the univariate and multivariate Cox proportional hazards regression analysis, we identified five independent prognostic factors in the training cohort. Age (≥65) at diagnosis (hazard ratio, HR = 2.722, *P* < 0.001), AJCC stage II (HR = 2.280, *P* = 0.023), AJCC stage III (HR = 6.090, *P* < 0.001), AJCC stage IV (HR = 21.310, *P* < 0.001), negative ER (HR = 2.956, *P* = 0.006), negative PR (HR = 1.825, *P* = 0.049), and no surgery (HR = 3.563, *P* < 0.001) were all significantly associated with BCSS in patients with MBC (Table 2). The related data for OS was in the Table S1.

**Table 2:** Univariate and multivariate Cox regression analysis based on all variables for cancer-specific survival (Training Cohort).

**Characteristics**	**Univariate analysis**		**Multivariate analysis**	
	**HR (95% CI)**	***P* value**	**HR (95% CI)**	***P* value**
**AGE**				
<65	Reference		Reference	
≥65	1.638 (1.077–2.489)	**0.021**	2.722 (1.720–4.306)	**<0.001**
**RACE**				
White	Reference		Reference	
Black	1.859 (1.183–2.921)	**0.007**	1.377 (0.845–2.244)	0.199
Other	1.231 (0.498–3.044)	0.652	0.907 (0.356–2.309)	0.838
**MARITAL STATUS**				
Married	Reference		Reference	
Unmarried	1.442 (0.963–2.159)	0.076	1.453 (0.940–2.248)	0.093
Unknown	1.040 (0.450–2.406)	0.926	1.473 (0.625–3.473)	0.376
**HISTOLOGY**				
Ductal	Reference		Reference	
Lobular	1.521 (0.212–10.926)	0.676	3.154 (0.430–23.158)	0.259
Mixed ductal and lobular	0.908 (0.224–3.686)	0.893	0.653 (0.157–2.722)	0.558
Others	1.479 (0.686–3.186)	0.318	1.558 (0.703–3.453)	0.275
**GRADE**				
I	Reference		Reference	
II	1.107 (0.489–2.509)	0.807	0.631 (0.270–1.477)	0.289
III	3.556 (1.632–7.748)	**0.001**	1.722 (0.752–3.941)	0.198
IV	–	0.976	–	0.996
**AJCC STAGE**				
I	Reference		Reference	
II	2.479 (1.238–4.965)	**0.010**	2.280 (1.120–4.639)	**0.023**
III	6.169 (3.091–12.312)	**<0.001**	6.090 (2.909–12.750)	**<0.001**
IV	31.747 (16.138–62.455)	**<0.001**	21.310 (9.698–46.815)	**<0.001**
**ER STATUS**				
Positive	Reference		Reference	
Negative	8.522 (4.746–15.303)	**<0.001**	2.956 (1.361–6.419)	**0.006**
**PR STATUS**				
Positive	Reference		Reference	
Negative	3.004 (1.862–4.846)	**<0.001**	1.825 (1.000–3.328)	**0.049**
**HER2 STATUS**				
Positive	Reference		Reference	
Negative	0.622 (0.370–1.047)	0.074	0.988 (0.565–1.728)	0.967
Borderline	0.208 (0.028–1.560)	0.127	0.336 (0.044–2.573)	0.294
**SURGERY**				
Performed	Reference		Reference	
Not performed	12.593 (8.011–19.795)	**<0.001**	3.563 (1.912–6.641)	**<0.001**
**RADIOTHERAPY**				
Yes	Reference		Reference	
No	0.933 (0.612–1.425)	0.749	1.351 (0.855–2.135)	0.197
**CHEMOTHERAPY**				
Yes	Reference		Reference	
No	0.688 (0.470–1.007)	0.055	0.971 (0.624–1.509)	0.894

*AJCC, The American Joint Committee for Cancer; ER, Estrogen receptor; PR, Progesterone receptor; HER2, Human epidermal growth factor 2-neu*.

*The bold values represent statistical significance*.

### Nomogram Construction

**Figure 1:**
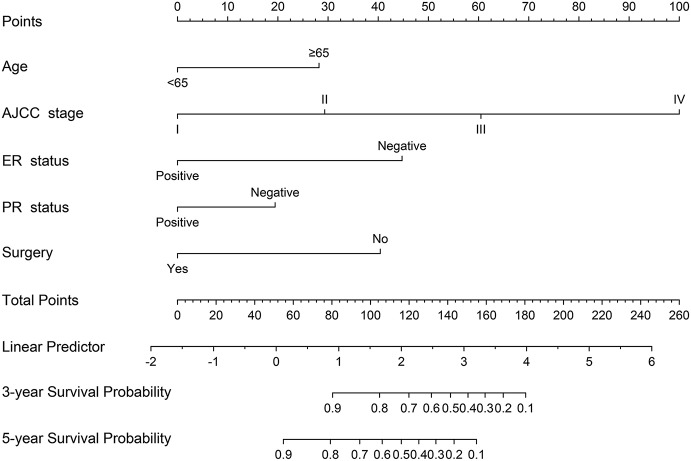
Nomogram predicted 3- and 5-year breast cancer-specific survival for male patients with five available factors, including age, the American Joint Committee for Cancer (AJCC) stage, estrogen receptor (ER) status, progesterone receptor (PR) status, and surgery.

A nomogram based on the selected prognostic factors from the training cohort was developed for the prediction of BCSS at 3 and 5 years (Figure 1). The nomogram demonstrated that AJCC stage contributed the most to prognosis, followed by ER status, surgery, age, and PR status. Each level of every variable was assigned a score on the points scale. By adding the scores for each of the selected variables, a total score was obtained. The prediction corresponding to this total score then helped in estimating the 3- and 5-year BCSS for each individual patient. The related results for OS were shown in the Figure S1.

### Validation and Calibration of the Nomogram

**Figure 2:**
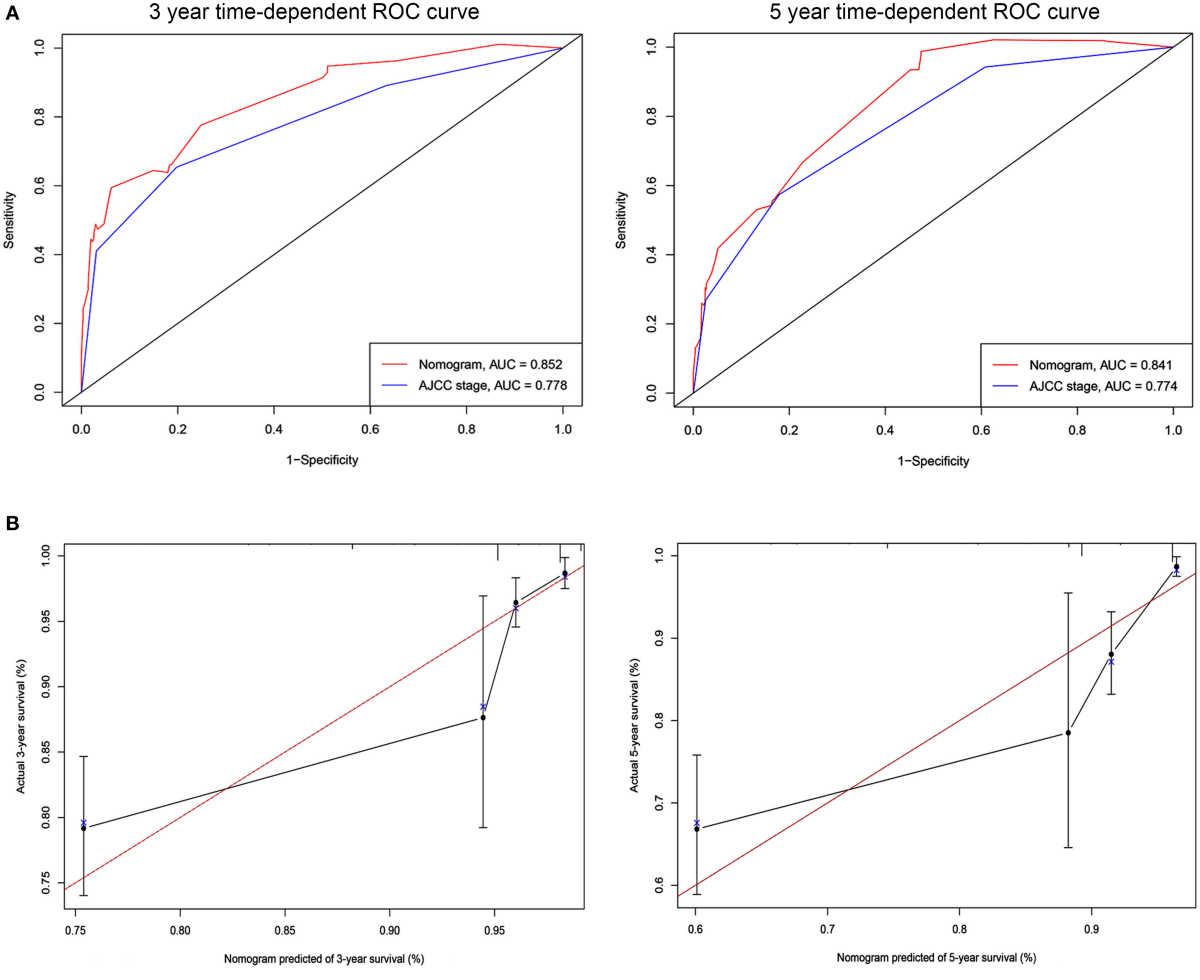
ROC curves and calibration plots for predicting patients-specific survival at 3- and 5-year in the training cohorts. **(A)** ROC curves of the Nomogram and AJCC stage in prediction of prognosis at 3- and 5-year point in the training set. **(B)** The calibration plots for predicting patient survival at 3- and 5-year point in the training set. ROC, receiver operating characteristic curve; AUC, areas under the ROC curve.

**Figure 3:**
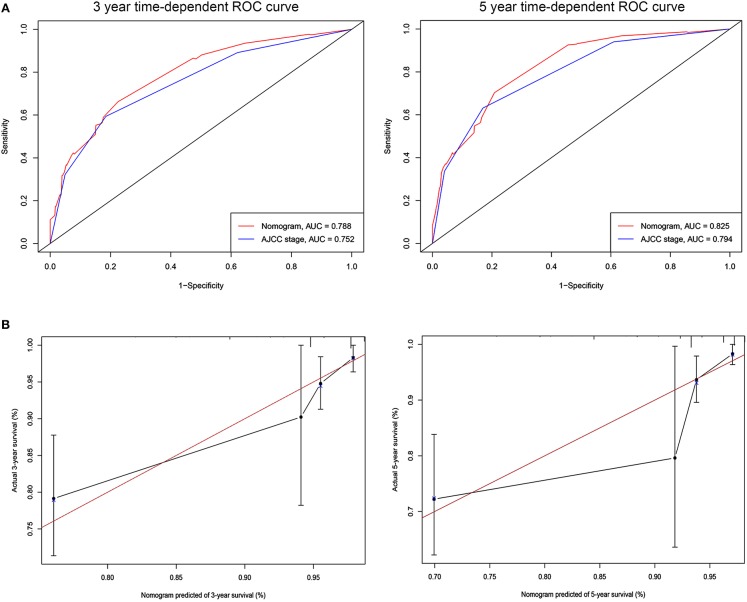
ROC curves and calibration plots for predicting patients-specific survival at 3- and 5-year in the validation cohorts. **(A)** ROC curves of the Nomogram and AJCC stage in prediction of prognosis at 3- and 5-year point in the validation set. **(B)** The calibration plots for predicting patient survival at 3- and 5-year point in the validation set. ROC, receiver operating characteristic curve; AUC, areas under the ROC curve.

The C-indices based on the nomogram (training group = 0.840, validation group = 0.818) were higher than those based on the AJCC stage (training group = 0.775, verification group = 0.768). Furthermore, our model demonstrated better discriminative ability compared to the traditional AJCC stage in both the training (3-year AUC: 0.852 vs. 0.778, 5-year AUC: 0.841 vs. 0.774, Figure 2A) and validation (3-year AUC: 0.778 vs. 0.752, 5-year AUC: 0.852 vs. 0.794, Figure 3A) cohorts for 3- and 5-year BCSS. The calibration plots of the nomogram showed good agreement between the actual observations and the predicted outcomes both in the training (Figure 2B) and validation (Figure 3B) cohorts for 3- and 5-year BCSS. The related results for OS were shown in the Figures S2, S3.

Analysis of accuracy showed that the NRI for the 3- and 5-year follow-ups were 0.193 (95% CI: 0.008–0.477) and 0.044 (95% CI: −0.036–0.437) in the validation cohorts, respectively. Similarly, in the validation set, the IDI for 3-and 5-years were 0.078 (*P* < 0.001) and 0.065 (*P* < 0.001), respectively. These results indicate that our nomogram has a greater potential for accurately predicting prognosis compared to the AJCC stage model.

**Figure 4:**
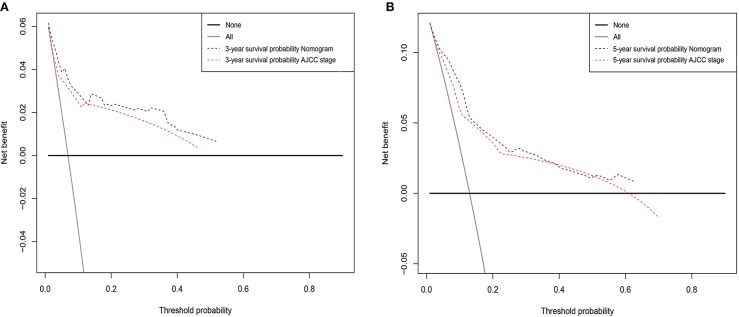
Decision curve analysis for the Nomogram and AJCC stage in prediction of prognosis of male patients at 3-year **(A)** and 5-year **(B)** point in the validation cohorts.

DCA was performed to compare the clinical usability and benefits of the nomogram with that of the traditional AJCC stage. As shown in Figure 4, compared to the AJCC stage model, the new nomogram's 3- and 5-year DCA curves showed larger net benefits across a range of death risk in the validation cohort.

## Discussion

MBC belongs to a small fraction of breast carcinomas and is usually diagnosed at an advanced stage. It has been found to have a worse prognosis compared to FBC (5, 13, 25). Therefore, it is necessary to establish a model to predict the risk for MBC, to aid the development of therapeutic strategies for these patients. Although the AJCC staging system is good for determining the prognosis in MBC patients (17), it neglects some significant risk factors such as age, race, and marital status. In the present study, we constructed a more comprehensive model based on a combination of various risk factors to better predict prognosis in MBC patients. This nomogram based on five variables including age, AJCC stage, ER status, PR status, and surgery was capable of making more accurate assessments and predictions in MBC patients compared to the traditional AJCC staging system both in the training and validation cohorts.

In our present investigation, we found that the proportion of male patients with negative ER, negative PR and radiotherapy were lower than the reported proportion of breast cancer in female cases (26, 27), and MC patients had a shorter median follow-up time than FBC patients (28). Additionally, we identified five risk factors that have an impact on the male breast cancer-specific survival (MBCCS). A previous study by Giordano et al. has shown that increasing age, black race, family history of breast cancer and radiation exposure can be risk factors for breast cancer in men (2). Nahleh et al. have reported that age, clinical stage, and lymph node status were independent prognostic factors for survival in MBC (29). Most of the studies have demonstrated that the median age at diagnosis of MBC is 68 years, which is 5–10 years older than that of women when diagnosed with breast cancer (12). Moreover, black men were at a higher risk of MBC than other races (30, 31). Also, MBC patients who had undergone surgery and radiotherapy have a better prognosis (31). The findings of our analysis are consistent with these previous reports.

Considering the influence of the above-mentioned risk factors, the traditional AJCC stage system might not predict survival well in MBC. We, therefore, developed a nomogram for predicting MBCSS by combining all of the effective factors. Consistent with findings in women with breast cancer (32), our model found that AJCC stage and ER status have a significant impact on the total score used for predicting the outcomes. The results showed that the C-index and calibration curve were good in the validation cohort, indicating that the model was reproducible and reliable. In our nomogram, negativity for ER and PR had higher risk scores implying that MBC patients who are negative for hormone receptors (HRs) had a poor prognosis, which is consistent with findings in FBC (33–35). However, it is noteworthy that the hormone levels are different for male and female patients, obviously due to the difference in sex. To the best of our knowledge, this is the comprehensive and intensive large-population study to construct a nomogram for patients with MBC.

We evaluated the value of this novel nomogram for predicting BCSS by comparing it to the traditional AJCC stage. Compared to the AJCC stage, our nomogram had better discriminability and accuracy for predicting 3- and 5-year BCSS. Additionally, using DCA, it was adequately proven that the established nomogram predicted survival with better accuracy than the AJCC staging system. Analogously, several studies have utilized DCA to verify the benefits and clinical utility of the predictive power of models (36, 37). This is the first study to compare a newly established model with the traditional AJCC staging model and demonstrate its better predictive ability for MBC patients. We believe our model will directly help clinicians quantify the risk of cancer specific death and thereby design appropriate therapeutic strategies for individual male patients.

Our study has some limitations that should be acknowledged. First, this is a large-sample retrospective study based on the SEER database, which may have some inherent biases. Second, several potential important parameters and specific information related to prognoses, such as the family history of breast cancer, the surgical margin status, vascular invasion, radiotherapy, and chemotherapy were not available in the SEER database. Third, we excluded patients who had missing data on the collected variables, which could have resulted in a selection bias. Forth, a small number of categorical variables classified according to common criteria, such as lobular carcinoma, grade IV, and metastatic sites, may lower the reliability of the findings. Finally, our nomogram was internally validated. It is important to evaluate it by external validation using other populations with MBC.

In conclusion, to better determine the prognosis in MBC patients, we constructed and validated a nomogram to predict 3-and 5-year BCSS based on a large, population-based cohort. The proposed nomogram considered five independent risk factors namely age, AJCC stage, ER status, PR status and surgery. We have confirmed the excellent discrimination and clinical usability of this nomogram by comparing it to the AJCC staging system.

## Ethics Statement

All of the authors signed the SEER Research Data Agreement in order to protect the patients' privacy, which is consistent with ethical principles.

## Author Contributions

SC and JL contributed to the conception and design. SC and YD analyzed the data. SC drafted the manuscript. YL, JY, QL, and HY contributed with a critical revision of the manuscript. All authors have read and approved the final version of the manuscript.

### Conflict of Interest Statement

The authors declare that the research was conducted in the absence of any commercial or financial relationships that could be construed as a potential conflict of interest.
