# Supplementary material for: Development and Validation of a Nomogram for Predicting Survival in Male Patients With Breast Cancer
Source: Front Oncol. 2019 May 14;9:361. doi: 10.3389/fonc.2019.00361 (PMC6527749; doi:10.3389/fonc.2019.00361)
Supplement: Supplementary file 1 [file Table_1.DOCX]

**Table S1 Univariate and Multivariate Cox regression analysis based on all variables for overall survival (Training Cohort)**

| **Characteristics** | **Univariate analysis** | | **Multivariate analysis** | |
| --- | --- | --- | --- | --- |
|  | **HR (95% CI)** | ***P* value** | **HR (95% CI)** | ***P* value** |
| **Age** |  |  |  |  |
| < 65 | Reference |  | Reference |  |
| ≥ 65 | 2.320 (1.751-3.074) | **< 0.001** | 2.648 (1.964-3.571) | **< 0.001** |
| **Race** |  |  |  |  |
| White | Reference |  | Reference |  |
| Black | 1.543 (1.132-2.104) | **0.006** | 1.155 (0.834-1.599) | 0.387 |
| Other | 0.582 (0.259-1.311) | 0.192 | 0.484 (0.213-1.098) | 0.082 |
| **Marital status** |  |  |  |  |
| Married | Reference |  | Reference |  |
| Unmarried | 1.290 (1.003-1.660) | **0.047** | 1.227 (0.943-1.596) | 0.128 |
| Unknown | 0.690 (0.375-1.270) | 0.233 | 0.771 (0.417-1.425) | 0.407 |
| **Histology** |  |  |  |  |
| Ductal | Reference |  | Reference |  |
| Lobular | 0.597 (0.084-4.254) | 0.606 | 0.894 (0.125-6.412) | 0.911 |
| Mixed ductal and lobular | 0.707 (0.263-1.900) | 0.492 | 0.644 (0.238-1.744) | 0.386 |
| Others | 1.660 (1.052-2.620) | **0.029** | 1.602 (0.999-2.570) | 0.051 |
| **Grade** |  |  |  |  |
| I | Reference |  | Reference |  |
| II | 1.241 (0.801-1.921) | 0.334 | 1.038 (0.666-1.618) | 0.870 |
| III | 1.991 (1.288-3.079) | **0.002** | 1.558 (0.990-2.452) | 0.055 |
| IV | - | 0.958 | - | 0.991 |
| **AJCC stage** |  |  |  |  |
| I | Reference |  | Reference |  |
| II | 1.372 (1.012-1.860) | **0.042** | 1.397 (1.021-1.912) | **0.037** |
| III | 2.028 (1.443-2.852) | **< 0.001** | 2.384 (1.639-3.467) | **< 0.001** |
| IV | 5.739 (3.900-8.445) | **< 0.001** | 4.068 (2.531-6.538) | **< 0.001** |
| **ER status** |  |  |  |  |
| Positive | Reference |  | Reference |  |
| Negative | 3.561 (2.112-6.005) | **< 0.001** | 2.349 (1.266-4.361) | **0.007** |
| **PR status** |  |  |  |  |
| Positive | Reference |  | Reference |  |
| Negative | 1.846 (1.300-2.622) | **0.001** | 1.419 (0.942-2.139) | 0.094 |
| **HER2 status** |  |  |  |  |
| Positive | Reference |  | Reference |  |
| Negative | 0.625 (0.451-0.866) | **0.005** | 0.669 (0.470-0.951) | **0.025** |
| Borderline | 0.754 (0.368-1.548) | 0.442 | 0.759 (0.365-1.579) | 0.460 |
| **Surgery** |  |  |  |  |
| Performed | Reference |  | Reference |  |
| Not performed | 7.055 (5.036-9.883) | **< 0.001** | 3.808 (2.489-5.827) | **< 0.001** |
| **Radiotherapy** |  |  |  |  |
| Yes | Reference |  | Reference |  |
| No | 1.178 (0.893-1.553) | 0.247 | 1.251 (0.923-1.697) | 0.149 |
| **Chemotherapy** |  |  |  |  |
| Yes | Reference |  | Reference |  |
| No | 1.324 (1.028-1.706) | **0.030** | 1.536 (1.147-2.057) | **0.004** |

AJCC: The American Joint Committee for Cancer; ER: Estrogen receptor; PR: Progesterone receptor; HER2: Human epidermal growth factor 2-neu.

**Figure legends**

**Figure S1** Nomogram predicted 3- and 5-year overall survival for male patients with sight available factors, including age, the American Joint Committee for Cancer (AJCC) stage, estrogen receptor (ER) status, human epidermal growth factor 2-neu (HER2) status, surgery and chemotherapy.

**Figure S2** ROC curves and calibration plots for predicting overall survival at 3- and 5-year in the training cohorts. (A) ROC curves of the Nomogram and AJCC stage in prediction of prognosis at 3- and 5-year point in the training set. (B) The calibration plots for predicting overall survival at 3- and 5-year point in the training set. ROC: receiver operating characteristic curve; AUC: areas under the ROC curve.

**Figure S3** ROC curves and calibration plots for predicting overall survival at 3- and 5-year in the validation cohorts. (A) ROC curves of the Nomogram and AJCC stage in prediction of prognosis at 3- and 5-year point in the validation set. (B) The calibration plots for predicting overall survival at 3- and 5-year point in the validation set. ROC: receiver operating characteristic curve; AUC: areas under the ROC curve.
