# Supplementary figures and images for: Development and Validation of a Nomogram for Predicting Survival in Male Patients With Breast Cancer
Source: Front Oncol. 2019 May 14;9:361. doi: 10.3389/fonc.2019.00361 (PMC6527749; doi:10.3389/fonc.2019.00361)

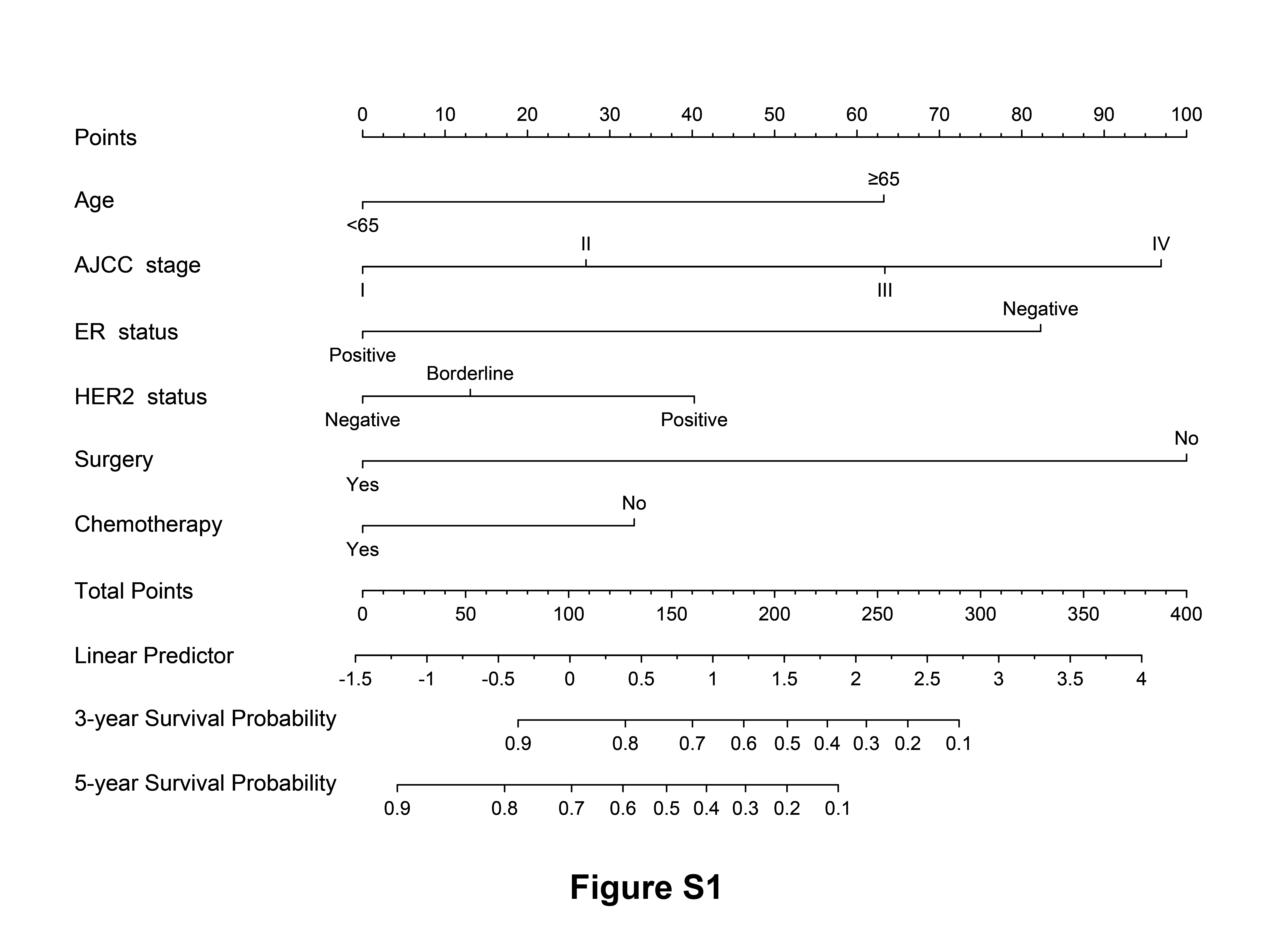

Supplement: Supplementary file 2 [file Image_1.TIF]

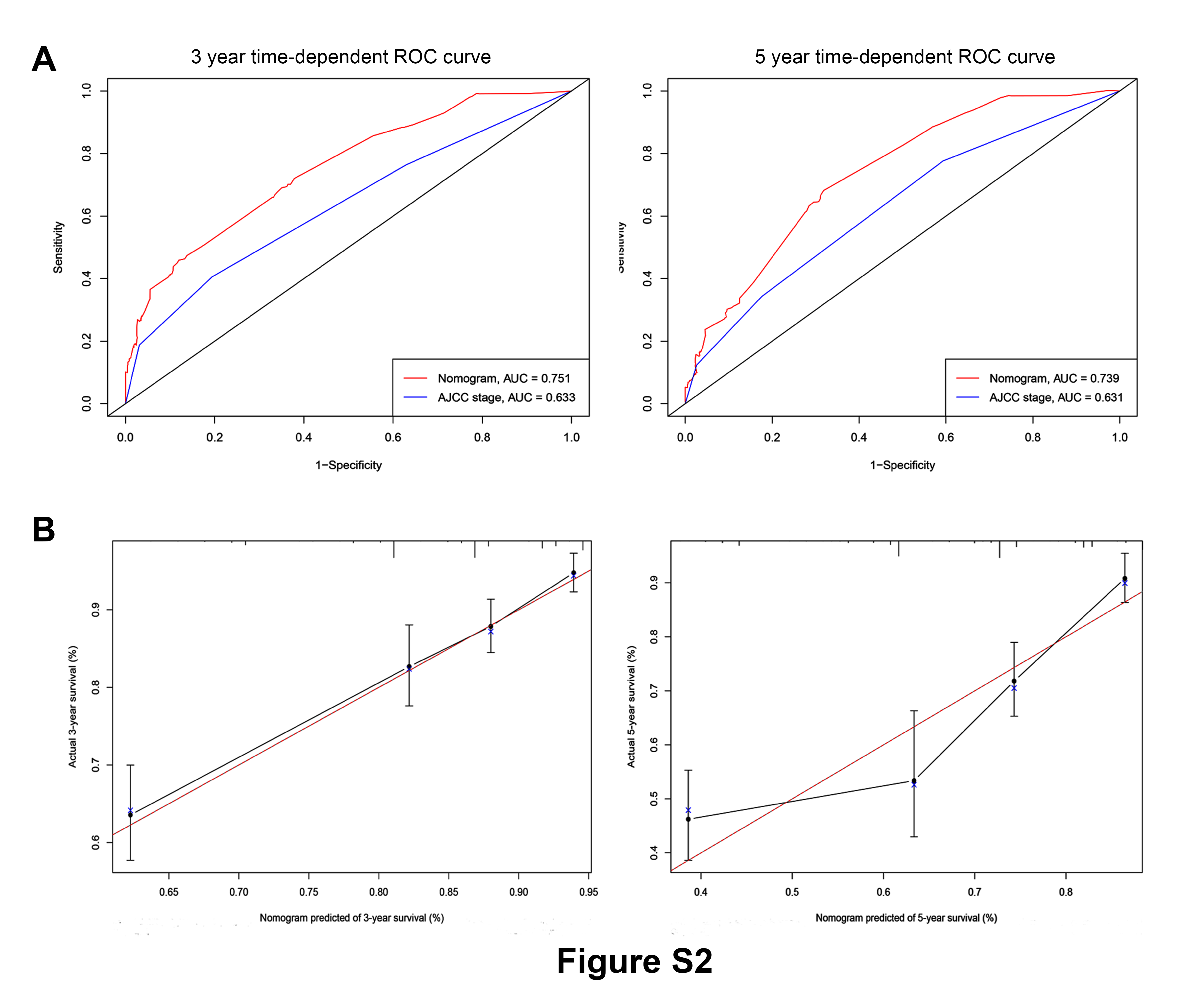

Supplement: Supplementary file 3 [file Image_2.TIF]

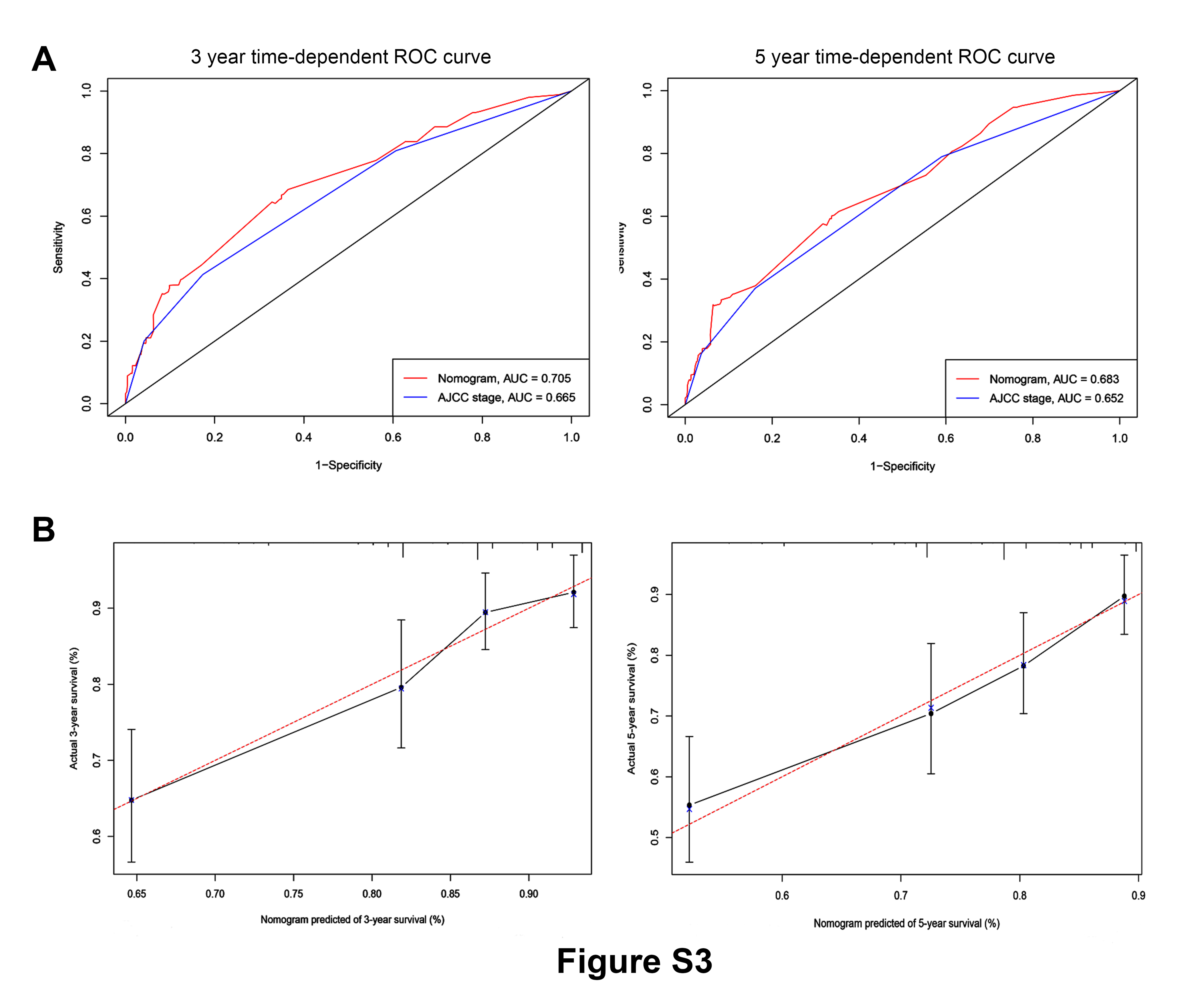

Supplement: Supplementary file 4 [file Image_3.TIF]
